# Antibacterial properties and urease suppression ability of *Lactobacillus* inhibit the development of infectious urinary stones caused by *Proteus mirabilis*

**DOI:** 10.1038/s41598-024-51323-0

**Published:** 2024-01-10

**Authors:** Dominika Szczerbiec, Katarzyna Bednarska-Szczepaniak, Agnieszka Torzewska

**Affiliations:** 1 Department of Biology of Bacteria, Faculty of Biology and Environmental Protection, University of Lodz, Banacha 12/16, 90-237 Lodz, Poland; 2 Laboratory of Medicinal Chemistry, Institute of Medical Biology PAS, Lodowa 106, 92-232 Lodz, Poland

**Keywords:** Microbiology, Diseases, Pathogenesis

## Abstract

Infectious urolithiasis is a type of urolithiasis, that is caused by infections of the urinary tract by bacteria producing urease such as *Proteus mirabilis*. *Lactobacillus* spp. have an antagonistic effect against many pathogens by secreting molecules, including organic acids. The aim of the study was to determine the impact of *Lactobacillus* strains isolated from human urine on crystallization of urine components caused by *P. mirabilis* by measuring bacterial viability (CFU/mL), pH, ammonia release, concentration of crystallized salts and by observing crystals by phase contrast microscopy. Moreover, the effect of lactic acid on the activity of urease was examined by the kinetic method and in silico study. In the presence of selected *Lactobacillus* strains, the crystallization process was inhibited. The results indicate that one of the mechanisms of this action was the antibacterial effect of *Lactobacillus,* especially in the presence of *L. gasseri*, where ten times less *P. mirabilis* bacteria was observed, compared to the control. It was also demonstrated that lactic acid inhibited urease activity by a competitive mechanism and had a higher binding affinity to the enzyme than urea. These results demonstrate that *Lactobacillus* and lactic acid have a great impact on the urinary stones development, which in the future may help to support the treatment of this health problem.

## Introduction

Urolithiasis is one of the most common diseases of the urinary system with an incidence of 1–13%, depending on the geographical region. It is reported that the number of cases and deaths from this highly widespread disease is constantly increasing, while the age of people suffering from urolithiasis is decreasing^1,2^. Many factors may be responsible for this trend, including diet, climate, physical activity or obesity^3^. In general, based on their chemical composition, urinary stones can be divided into calcium oxalate stones (which are the most common type), calcium phosphate stones, uric acid stones, cystine stones and struvite stones. The last type includes stones, which are formed as a result of the infection in the urinary tract^4^. According to the literature data, infectious stones account for even 15% of all urinary stones^5–7^ and they are caused by the activity of bacterial urease, a nickel-dependent metalloenzyme^8^. Microorganisms responsible for this process belong mainly to the genus *Proteus*. They are isolated from up to 70% of infectious stones^9^ and all of *Proteus* isolates from urinary stones produce urease^7,10^. Urease catalyzes the hydrolysis of urea into carbon dioxide (CO_2_) and ammonia (NH_3_), which increases urinary pH. The concentrations of ammonium, bicarbonate and phosphate ions increase, which in the presence of magnesium and calcium ions, leads to the precipitation of carbonate apatite (Ca_10_(PO_4_)_6_CO_3_) and struvite (MgNH_4_PO_4_·6H_2_O)^5^. Precipitation of mineral components of urine caused by their excessive concentration in relation to the solubility leads to crystallization (struvite and apatite crystals) –the initial stage of urinary stones formation. During the next phase, the struvite and apatite crystals aggregate, which results in the formation of a urinary stone and its retention in the urinary tract^11^.Treatment of infectious urolithiasis is a long-term and complicated process. It includes antibiotic treatment to eliminate the pathogen, stone removal using shock wave lithotripsy (SWL) or percutaneous nephrolithotomy (PCNL) and recurrence prevention^10,12^. Antibiotic therapy is often challenging. An antibiotic is not able to penetrate into the stone, where microorganisms can also live, which allows them to survive and leads to the formation of urinary stones de novo^13^. There are several methods of supporting the urolithiasis treatment e.g. use of urease inhibitors or urinary acidification with ascorbic acid, ammonium chloride, ammonium sulfate, ammonium nitrite and l-methionine^10,12^. Nevertheless, due to the lack of proper methods of prevention and the fact that current treatment does not prevent the recurrences, there is a need to find alternative sources that will act preventively or may support the traditional treatment of the developing disease.

According to many data*, Lactobacillus* belong to the natural microbiota of the urinary tract have a huge impact on this environment and uropathogens^14–16^. They exert their protective properties through many mechanisms like producing antibacterial compounds (bacteriocins, hydrogen peroxide, organic acids), inhibition of pathogen adhesion or exhibiting immunomodulatory effect. Recently, there have been many indications of the possibility of using lactic acid bacteria for the treatment and prevention of UTI^17–19^. Many recent in vitro studies also emphasized the use of probiotic strains, including *Lactobacillus*, to treat or prevent the development of calcium oxalate stones^20,21^. Therefore, it is interesting to know if natural microbiota of the urinary tract has also an influence on one of the UTIs complications i.e. formation of infectious urinary stones.

Our study focused on the interactions between the natural microbiota of the human urinary tract and *Proteus*, one of the genera, most frequently isolated from infectious urinary stones. The present paper was designed to determine the impact of *L. crispatus, L. jensenii* and *L. gasseri* isolated from human urine and substances secreted by them on the inhibition of the crystallization process caused by *P. mirabilis* and on urease activity, which plays an important role in the development of infectious urinary stones.

## Material and methods

### Bacterial strains

*P. mirabilis* strains (KP; 5628) were isolated at the Department of Microbiology from the urine of patients of the Children’s Memorial Health Institute in Warsaw, Poland, who had been diagnosed with infectious urolithiasis. The other two *P. mirabilis* strains (608/221; K8/MC) were obtained from urinary stones and provided by the Provinicial Specialist Hospital M. Pirogow in Lodz. The strains were identified using the API 20E test (Biomerieux, Marcy-I’Etoile, France) and cultured on TSB (tryptic soy broth, BTL, Warsaw, Poland) for 24 h at 37 °C.

*Lactobacillus* strains were isolated from the urinary tract of healthy people, and were deposited in the bacterial strain collection at the Department of Biology of Bacteria, University of Lodz. The method of isolation of these strains and their characteristics had been described in our previous study^14^. Briefly, strains were obtained from human urine, of both men and women who had not been treated with antibiotics or probiotics in the last 3 months. Urine samples were obtained from all participants and/or legal guardians with their informed consent and the research were carried out in accordance with relevant guidelines and regulations. All experimental protocols were approved by University of Lodz Research Ethics Committee (approval number 4/(I)/KBBN-UŁ/II/2020). Strains, were identified by mass spectrometry MALDI/TOF Microflex LT (Bruke, Billerica, MA, USA). *Lactobacillus* spp. were cultured on APT agar (BD Difco, Franklin Lakes, NJ, USA) and incubated in 5% CO_2_ at 37 °C for 48 h.

### Synthetic urine

Synthetic urine, the composition of which chemically corresponds to the mean concentrations found in normal human urine during a 24-h period was made as described by Griffith et al.^22^ with some modifications. It contained the following components [g/L]: CaCl_2_ × 2H_2_O, 0.651; MgCl_2_ × 6H_2_O, 0.651; NaCl, 4.6; Na_2_SO_4_, 2.3; sodium citrate 0.65; sodium oxalate 0.02; KH_2_PO_4_, 2.8; KCl, 1.6; NH_4_Cl, 1.0; urea 25.0; creatinine, 1.1 and tryptic soy broth, 10.0 (Sigma, St. Louis, MO, USA). The solution was prepared immediately before the experiment and sterilized by passing through a 0.2 µm pore-size filter (Sartorius, Goettingen, Germany).

### Crystallization experiment in mixed cultures

The crystallization assay was performed according to the Torzewska et al. method^23^ with some modifications. To 20 mL of synthetic urine, 20 µL of bacterial cultures were added in the ratio 1:5 of *P. mirabilis* (KP, K8/MC, 5628, 608/221) and *Lactobacillus* (*L. crispatus* 1.2, *L. crispatus* 4, *L. jensenii* 22.2 and *L. gasseri* 35.3) (it was determined that in this ratio, crystallization was most intensively inhibited by *Lactobacillus*, data not shown). *Lactobacillus* and *Proteus* strains were cultured as described in section “Bacterial strains”.

At first, the induction time of crystallization i.e., the time between the creation of supersaturation and the appearance of crystals was assessed. The absorbance of the above samples was measured every half hour at a wavelength of 600 nm using a spectrophotometer (Ultraspec 2000, Pharmacia Biotech, USA). It allowed selection of crucial hours in which the intensity of crystallization was determined.

At 0, 3, 6, 8 and 24 h, the pH, the number of *P. mirabilis*, the amount of released ammonia, and the intensity of crystallization were assessed. Pure cultures of *P. mirabilis* in synthetic urine were controls in this experiment. Confirmation of the crystallization of struvite and apatite occurring under such experimental conditions was carried out in our previous studies^24,25^. The intensity of crystallization was assessed on the basis of quantitative and qualitative determinations. Quantitative research included the assessment of the Ca^2+^ and Mg^2+^ ions concentrations by atomic absorption spectroscopy (SpectAA-300 Varian, Palo Alto, California). For these analyses, 1 mL of each sample was collected, centrifuged (8000 rcf, 5 min), and treated for mineralization with 0.5 mL 65% HNO_3_. Struvite and apatite crystals were observed using a phase-contrast microscope (Nikon Eclipse TE-2000-S). The pH was determined using a pH meter (Elmetron Cp-215, Zabrze, Poland) and the ammonia release was measured by the phenol hypochlorite colorimetric method^26^. The results were expressed as % of inhibition of ammonia release, where 100% was the concentration of ammonia in the control, synthetic urine with *P. mirabilis*. The number of *P. mirabilis* was determined by spreading 100 µL of serially diluted suspensions on TSB agar with 0.1% phenol. Grown colonies were counted after 24 h incubation at 37 °C, to determine the number of CFU/mL in mixed and pure cultures. In addition, at the beginning of the assay the number of *Lactobacillus* was determined by spreading the tested samples on APT agar to establish the ratio of *Proteus* to *Lactobacillus* (1:5).

### Determination of crystallization and urease activity in the presence of *Lactobacillus* and lactic acid

The crystallization micromethod allowed examining the impact of *Lactobacillus* strains and lactic acid produced by them on the crystallization process and on the activity of the urease enzyme. The degree of crystallization was assessed using phenol red as a pH indicator, due to the fact that an increase in pH indicates the start of the crystallization process. At first, the influence of four tested *Lactobacillus* (*L. crispatus* 1.2, *L. crispatus* 4, *L. jensenii* 22.2 and *L. gasseri* 35.3) on crystallization caused by *P. mirabilis* strains was assessed according to the Torzewska et al. method^23^. 150 µL of synthetic urine with 0.001% phenol red was added to 96-well plates with 2 µL of *P. mirabilis* (2 × 10^8^ CFU/mL) and *Lactobacillus* cultures in proportion 1:5 (*P. mirabilis*:*Lactobacillus*). *Lactobacillus* and *Proteus* strains were cultured as described in section "Bacterial strains". Negative control was 150 µL of synthetic urine with 0.001% phenol red and positive control was pure *P. mirabilis* cultures. All wells were covered with mineral oil to prevent the release of ammonia and increase the pH in the adjacent wells, and incubated for 24 h at 37 °C. The absorbance was measured using a microplate reader Multiskan Ex (Labsystems, Helsinki, Finland) at a wavelength of 550 nm. The impact of *Lactobacillus* strains on the activity of urease from Jack bean (Serva, Heidelberg, Germany) was performed similarly. 190 µL of synthetic urine with 0.001% phenol red was added to 96-well plates with 1 µL of the urease enzyme (at the final concentration 0.105 U/mg) and 10 µL of *Lactobacillus* cultures. Positive control was synthetic urine with urease and negative control was synthetic urine. The plate was incubated at 37 °C, and every hour, up to 6 h of incubation, the absorbance was measured at a wavelength of 550 nm. The ammonia release inhibition was measured as described in section "Crystallization experiment in mixed cultures". The effect of different lactic acid (Sigma, St. Louis, MO, USA) concentrations (1.4 mM, 2.8 mM 5.5 mM, 11 mM, 22 mM) on urease was assessed using the same method. Briefly, 190 µL of synthetic urine with 0.001% phenol red was added to 96-well plates with 1 µL of urease and 10 µL of dilutions of lactic acid*.* Positive and negative controls were the same as in the assay described above. The plate was incubated at 37 °C and the absorbance was measured at a wavelength of 550 nm.

### Urease inhibition assay

The assay was performed to determine the mechanism of urease inhibition by lactic acid. The method was based on the research conducted by Rashid et al.; Tan et al. and Du et al.^27–29^ with our modifications. The assay was carried out in a phosphate buffer containing 5.9 mM EDTA and 25 mM HEPES (pH 8.0) in 96-well plates. The enzymatic mixture contained: 150 μL of urea solutions (Chempur, Piekary Śląskie, Poland) in different concentrations (5 mM, 10 mM, 15 mM, 30 mM, 45 mM), 40 µL of urease from the Jack bean enzyme (Serva, Heidelberg, Germany) at the final concentration 0.105 U/mg and 10 µL of lactic acid (final concentrations 11 mM, 38 mM, 55 mM, dissolved in distilled water). Distilled water was added to control samples instead of lactic acid. The plate was incubated at 37 ºC at time points: 2, 5, 10, 30, 60 and 120 min, the amount of released ammonia was determined using the phenol-hypochlorite colorimetric method^26^. The absorbance was measured after 30 min using a microplate reader Multiskan Ex (Labsystems, Helsinki, Finland) at a wavelength of 620 nm. The IC_50_ of lactic acid was determined for the 0.105 U/mg concentration of urease and 10 mM of urea in the concentration range of lactic acid from 110 to 1 mM. The IC_50_ value of lactic acid was calculated using a GraphPad Prism 8.0 (GraphPad Prism Software Inc., San Diego, CA, USA) for the dose–response curve.

### Docking study

#### Homology modeling of Proteus mirabilis urease

The sequence of the alpha subunit of *P. mirabilis* urease was obtained from UniProtKB Database^30^ (URE1_PROMH P17086). Top-ranked templates were selected in a SwissModel homology-modeling server^31^ and obtained from the Protein Data Bank (PDB). The crystallographic structure of Jack bean (*Canavalia ensiformis*) urease was identified as the best-matching template based on HHBlits^32^. (PDB 4gy7.1.A; resolution 1.49 Å, sequence identity 60.6%, sequence similarity 0.48). In the SwissModel homology-modeling, the model of *P. mirabilis* urease was built by template alignment method using ProMod3 3.2.1 Version 3.2.1^33^. In ProMod3, biologically relevant non-covalently bound ligands are considered in the model if they have at least three coordinating residues in the protein, those residues are conserved in the target–template alignment and the resulting atomic interactions in the model are within the expected ranges. In our model, the interaction of amino acids at the catalytic site, including His residues, and Ni ion ligands was found to be conserved between the target and the template. Therefore, Ni ions in the template structure were identified as relevant ligands and transferred by homology to the model. Finally, the resulting model's geometry was regularized using a force field by ProMod3 tools.

The global and per-residue model quality has been assessed using the QMEAN scoring function^34^. Global model quality estimate GMQE was 0.88, QMEANDisCo global was 0.84 ± 0.05, QMEAN Z-score -0.97. Predicted local similarities of model and target histidine residues at the catalytic site (QMEANDisCo Local) were 0.93 for His346B, 0.93 for His272B, 0.93 for His134B, and 0.86 for His136B. The final model structure was pre-processed and optimized in Maestro Schrodinger 11.7 (Schrödinger, Inc., New York, NY, 2013). The reference structure of the Jack bean urease (.pdb file 4gy7.1) was also prepared in the Maestro Schrodinger 11.7 software for docking comparison.

#### Preparation of ligands

The 3D structures of L( +) lactic acid and urea were obtained in the form of .sdf files from the PubChem database (CID:612 and CID:1176, respectively^35^, optimized in MarvinSketch 20.14.0, 2020, ChemAxon (http://www.chemaxon.com) and calculated at the DFT/B3LYP/6-31G* level (HyperChem 7.51, HyperCube Inc., Gainesville, FL, USA). Partial charges were preserved in docking experiments.

#### Docking experiments

Nickel  2+ ion parameters were implemented into the AutoDock 4.2.6 database and to the AutoGrid parameter files: vdW diameter, Rii 1.41 Å^36^ and vdW well depth, epsii 0.013 kcal/mol^37^; the remaining parameters were default as for metal ions. Kollman charges were assigned to protein atoms, charge + 2 to Ni ions. A docking area was centered at the average position of the intra- and extracellular part of a protein broadly covered catalytic center and external amino acids (80 × 80 × 80 box, grid point spacing 0.375 Å). The Lamarckian genetic algorithm was used for conformational search of a flexible ligand. Docking parameters were as follows: number of individuals in population 100, GA-LS runs 50, maximum number of energy evaluations 2.5 × 10^8^, maximum number of generations 27,000. The ligand docking poses were analyzed and visualized using the Protein–Ligand Interaction Profiler (PLIP 2.2.0 open source software)^38^ and PyMOL Molecular Graphics System, Version 2.3.4, Schrödinger, LLC.

### Statistical analysis

All experiments were carried out at least in triplicate. Statistical analyses were based on the Kruskal–Wallis test and the Mann–Whitney U test, performed using TIBCO Software Inc. (2017). Statistica (data analysis software system), version 13. http://statistica.io. The results were considered to be statistically significant at p value < 0.05.

## Results

### The effect of *Lactobacillus* strains on the intensity of crystallization caused by *P. mirabilis* strains in vitro

#### Changes in urinary pH and in a degree of ammonia release

**Figure 1:**
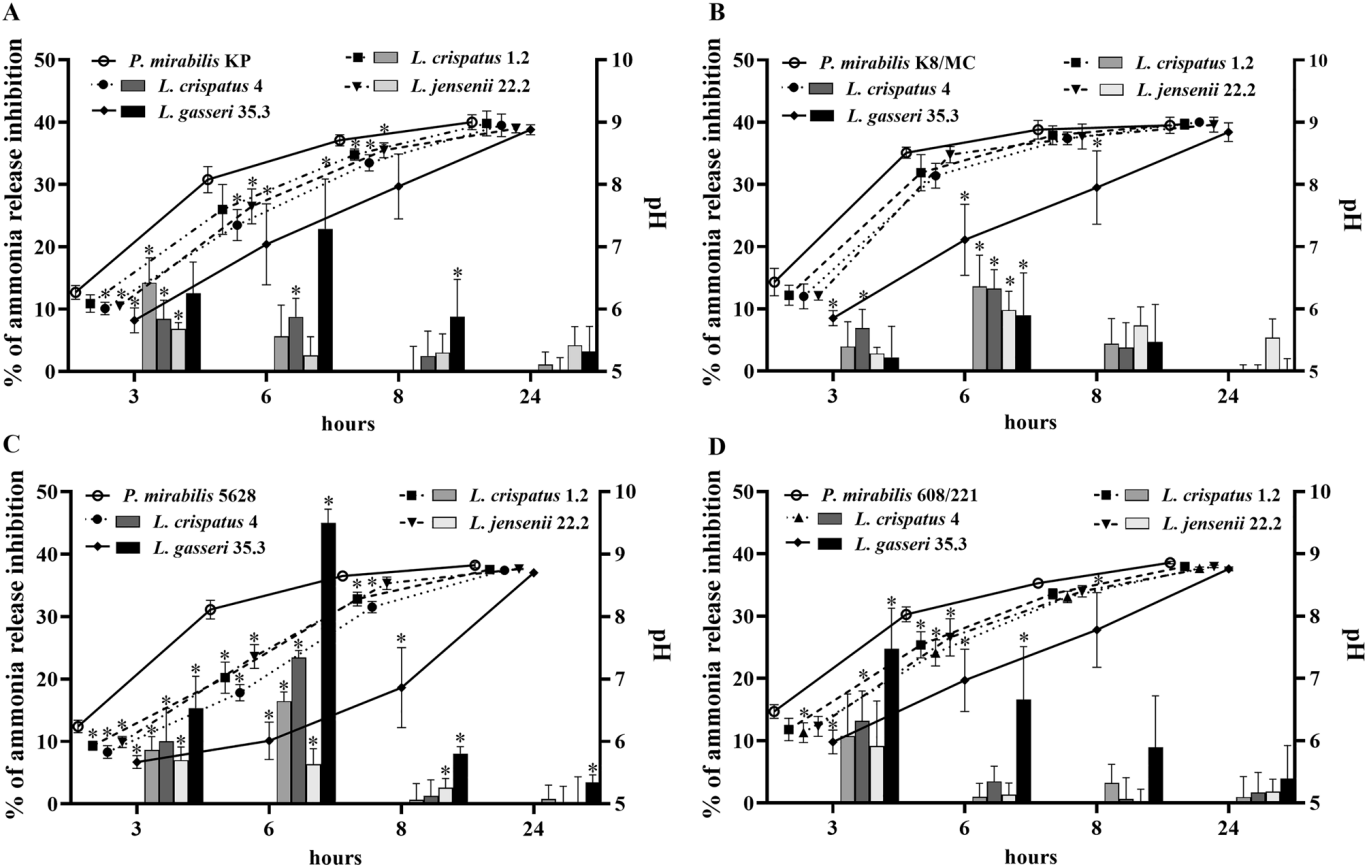
Percentage of ammonia release inhibition (bars) in mixed cultures in synthetic urine, where pure *P. mirabilis* samples were 100%, and changes in urinary pH (lines) in pure and mixed samples after 3, 6, 8 and 24 h of incubation. (**A**) Corresponds to *P. mirabilis* KP; (**B**) *P. mirabilis* K8/MC; (**C**) *P. mirabilis* 5628 and (**D**) *P. mirabilis* 608/221. The results are presented as mean ± standard deviation (SD) of three experiments; *p < 0.05 for comparison of the pH value and ammonia release of *P. mirabilis* pure culture vs. co-culture with *Lactobacillus*, Mann–Whitney U test.

At the beginning of the experiment urinary pH in pure and mixed samples averaged 5.7 (data not shown). During the incubation the pH in those samples constantly increased but with a different pattern. At every hour of the experiment (except 24 h) the lowest pH (compared to the controls) was observed in the sample with *L. gasseri* 35.3. Significantly lower pH values in the tested samples were noticeable mainly in the sixth hour of incubation, whereas after 24 h the pH values were close to each other and averaged 9.0. The level of released ammonia was closely related to the pH level. The ammonia released by the action of the urease enzyme, increased the urinary pH. In Fig. 1. we can observe that in the presence of *L. gasseri* 35.3 the ammonia release was lower compared to the control. In particular, after 6 h of incubation, it was inhibited from 10% for *P. mirabilis* K8/MC to even 45% for *P. mirabilis* 5628.

#### Viability of *P. mirabilis* in pure and mixed cultures

**Figure 2:**
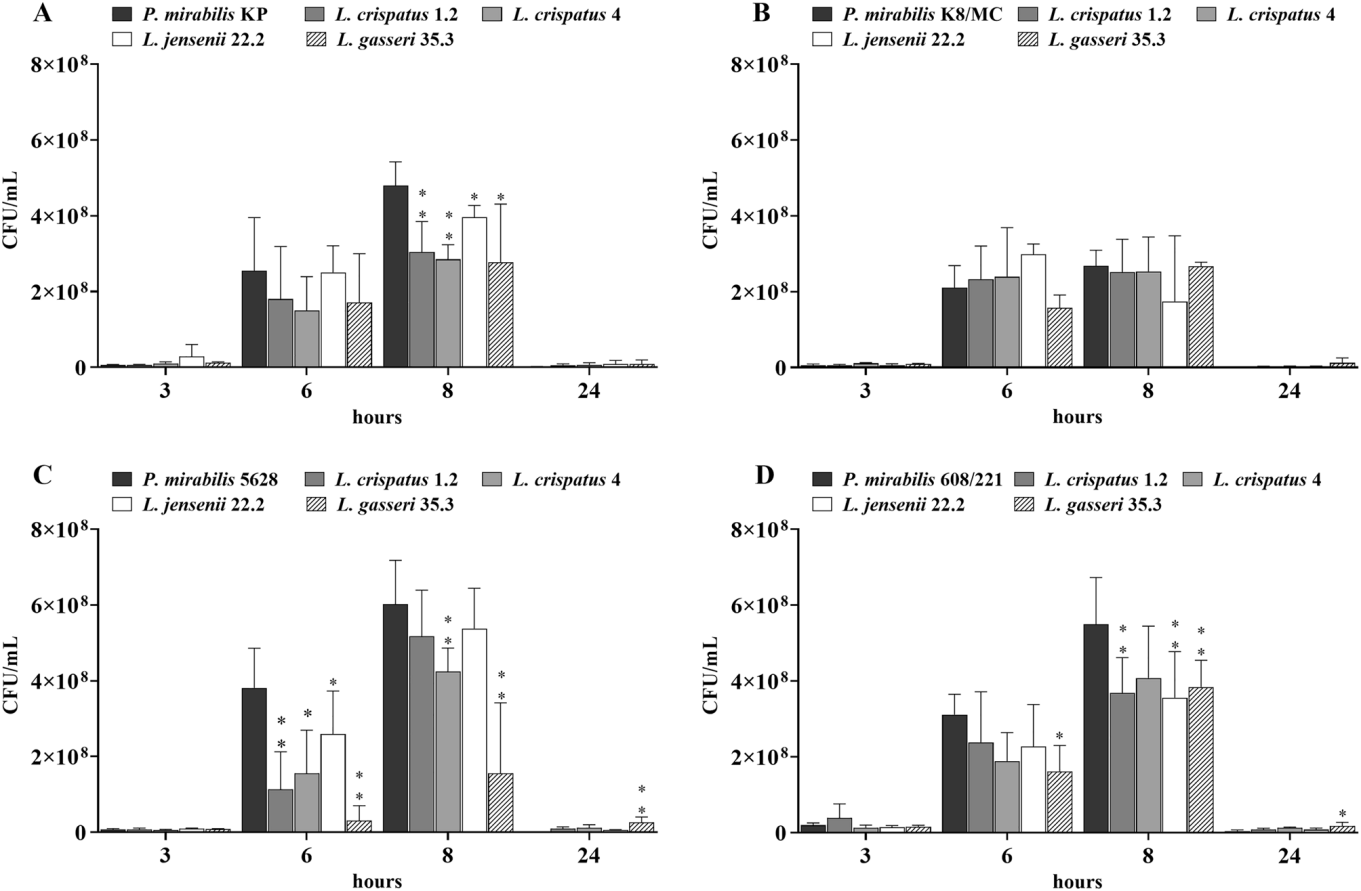
*P. mirabilis* viability after 3, 6, 8, and 24 h of incubation in pure and mixed cultures in synthetic urine. (**A**) corresponds to *P. mirabilis* KP; (**B**) *P. mirabilis* K8/MC; (**C**) *P. mirabilis* 5628 and (**D**) *P. mirabilis* 608/221. The results are presented as mean ± standard deviation (SD) of three experiments, **p < 0.01, *p < 0.05 for *P. mirabilis* viability in mixed cultures vs. pure culture, Mann–Whitney U test.

At 0, 3, 6, 8, and 24 h of the assay, the number of *P. mirabilis* bacteria was determined. During the first three hours of the experiment, no significant changes in *Proteus* viability were observed in mixed cultures compared to the control. As shown in Fig. 2, after 6 h of incubation, the number of *P. mirabilis* bacteria in control samples reached a value of 2–4 × 10^8^ CFU/mL. However, in some tested samples with *Lactobacillus* strains the growth inhibition of *P. mirabilis* strains was observed. All tested lactobacilli significantly inhibited *P. mirabilis* 5628 growth, especially in the presence of *L. gasseri* 35.3, 10 times less *P. mirabilis* 5628 bacteria were observed, compared to control (**p < 0.01). The same effect was noted in the sample with *P. mirabilis* 608/221. Inhibition of *Proteus* viability after 6 h was not observed in the remaining samples (*P. mirabilis* KP and K8/MC). It is worth mentioning that in the sample with *P. mirabilis* 5628, the inhibition of viability by the *L. gasseri* strain was still maintained after 8 h of incubation and the same effect was noted in co-culture of *P. mirabilis* KP and 608/221 with *L. crispatus *or *L. gasseri*. After 24 h the viability of *P. mirabilis* strains decreased in all samples and averaged 5 × 10^6^ CFU/mL. Only in co-culture with *L. gasseri*, it was observed that some of the tested *P. mirabilis* strains showed greater viability than in control during this hour.

#### Intensity of crystallization

**Figure 3:**
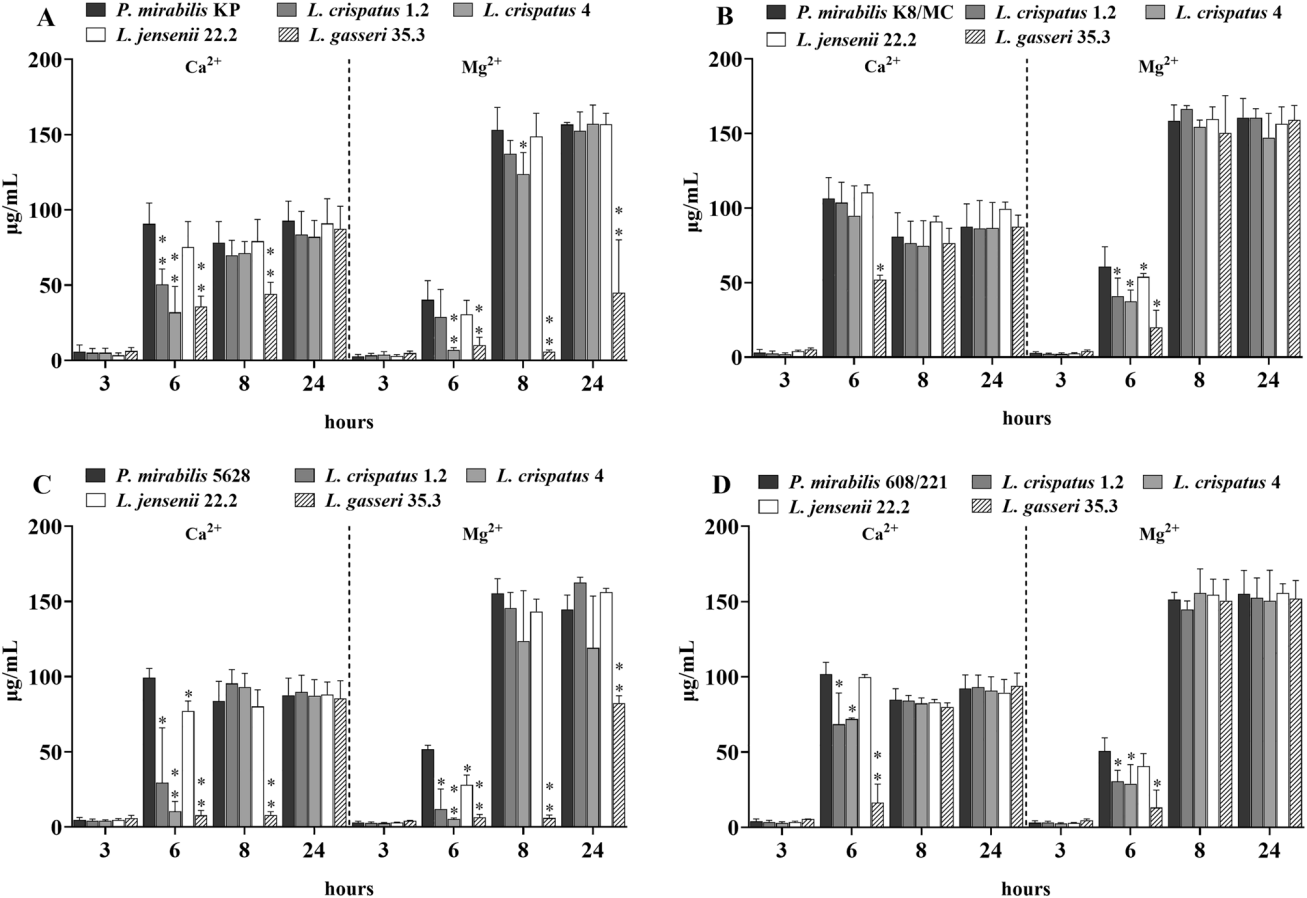
Calcium and magnesium concentrations in the tested and control samples after 3, 6, 8 and 24 h of incubation in synthetic urine. (**A**) Corresponds to *P. mirabilis* KP; (**B**) *P. mirabilis* K8/MC; (**C**) *P. mirabilis* 5628 and (**D**) *P. mirabilis* 608/221. The results are presented as mean ± standard deviation (SD) of three experiments, **p < 0.01, *p < 0.05 for *P. mirabilis* viability in mixed cultures vs. pure culture, Mann–Whitney U test.

Intensity of crystallization in the tested samples was assessed quantitatively and qualitatively. Quantitative analyses showed that in the first three hours no significant changes in the concentration of the tested ions were observed. Nevertheless, after 6 h of incubation the most significant differences were observed between the amount of Mg^2+^ and Ca^2+^ ions in the tested samples compared to the controls (pure *P. mirabilis* cultures in synthetic urine) (Fig. 3). A significant decrease in the amount of these two ions was noted especially in the presence of the *L. gasseri* strain, in contrast to *L. jensenii* which in most cases did not exhibit such properties. This trend was the greatest in co-culture with *P. mirabilis* 5628 and *L. gasseri*, where the calcium concentration was even 10 times lower than in the control, and the amount of magnesium was up to 5 times lower after 6 h. In the presence of *L. gasseri*, a lower content of the tested ions was still observed after eight hours of incubation in co-culture with *P. mirabilis* KP and 5628. The low content of Mg^2+^ was maintained in those samples after 24 h. It is worth noting that from the eighth hour, the proportion of calcium and magnesium in the samples started changing, and higher concentrations of Mg^2+^, the main ion of struvite crystals were observed.

**Figure 4:**
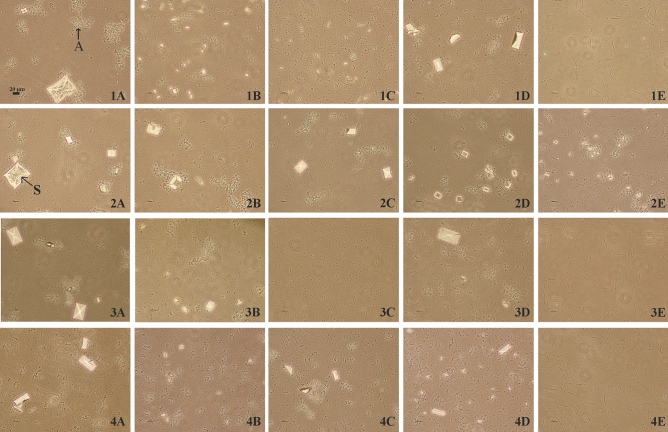
Carbonate apatite (A) and struvite (S) crystals in mixed and pure cultures after 6 h of incubation. (**1**) corresponds to *P. mirabilis* KP; (**2**) *P. mirabilis* K8/MC; (**3**) *P. mirabilis* 5628 and (**4**) *P. mirabilis* 608/221 and (**A**) control samples; (**B**) samples with *L. crispatus* 1.2; (**C**) *L. crispatus* 4; (**D**) *L. jensenii* 22.2 and (**E**) *L. gasseri* 35.3. The scale bar represents 20 µm.

Observation with a phase-contrast microscope enabled a qualitative assessment of the formation of carbonate apatite and struvite crystals (Fig. 4). After 6 h, in all tested samples crystals formed in the crystallization process were smaller compared to the control or even absent, mainly in the samples with *L. gasseri* 35.3. All crystals showed „coffin-lid”-shaped appearance. The largest of struvite crystals were observed in control samples (even 90 µm width). After 8 and 24 h of incubation, crystals were found in all samples, but they were still smaller compared to the controls.

### The impact of *Lactobacillus* strains and lactic acid on crystallization and urease activity

**Figure 5:**
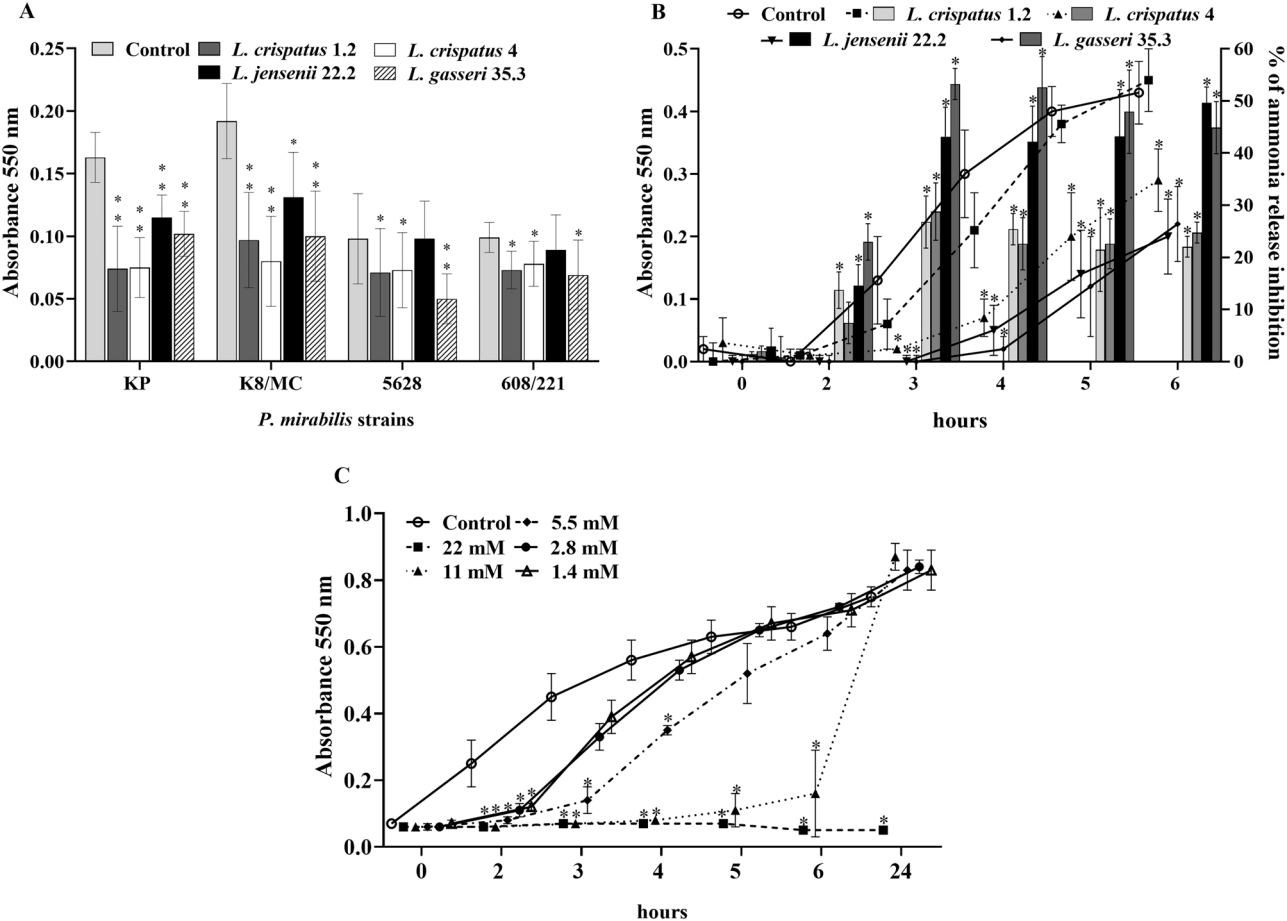
**(A**) The effect of *Lactobacillus* strains on crystallization in synthetic urine with phenol red caused by four tested *P. mirabilis* strains after 5 h of incubation in the ratio 1:5. (**B**) The effect of *Lactobacillus* strains on crystallization in synthetic urine with phenol red caused by the Jack bean urease (lines) and % of ammonia release inhibition (bars) in samples with *Lactobacillus* compared to the control with urease alone. (**C**) The effect of different concentrations of lactic acid on crystallization in synthetic urine with phenol red. The results are presented as mean ± standard deviation (SD) of three experiments; **p < 0.01, *p < 0.05 for comparison of controls vs. tested samples, Kruskal–Wallis test.

As shown in Fig. 5A. *Lactobacillus* strains inhibited the process of crystallization caused by *P. mirabilis* strains. However, the degree of inhibition varied in the presence of different strains. *L. jensenii* 22.2 exhibited the lowest inhibition ability, while the others were found to be effective in suppressing the crystallization of all *P. mirabilis* strains. On the other hand, *Proteus* strains also showed different levels of effectiveness in this process. We distinguished strains KP and K8/MC as those exhibiting the strongest ability of urine components crystallization, as opposed to strains 5628 and 608/221. The assay with the Jack bean urease enzyme brought a lot of crucial data about the influence of *Lactobacillus* strains on the activity of this enzyme. Figure 5B (lines) shows that all *Lactobacillus* strains inhibited the enzyme activity but with different intensity. *L. gasseri* constantly caused the highest level of crystallization inhibition compared to the control and this strain also inhibited the release of ammonia the most intensively (bars). One of the main extracellular substances with antagonistic activity which are secreted by *Lactobacillus* spp. is lactic acid. Regarding this, we also investigated the impact of this acid on urease activity. The results are shown in Fig. 5C. different concentrations of lactic acid inhibited crystallization, and it was observed that with the increase in the acid concentration, the inhibition process was also intensified. Lactic acid at the concentration of 22 mM suppressed crystallization most effectively, and in its presence no increase in crystals formation was observed even after 24 h. However, the lowest concentrations (2.8 mM, 1.4 mM) inhibited the process only up to about 4 h of the experiment. The obtained results show that lactic acid produced by the tested *Lactobacillus* strains is able to inhibit the crystallization process and the level of inhibition is related to the concentration of the acid.

### Mechanism of urease inhibition by lactic acid

**Figure 6:**
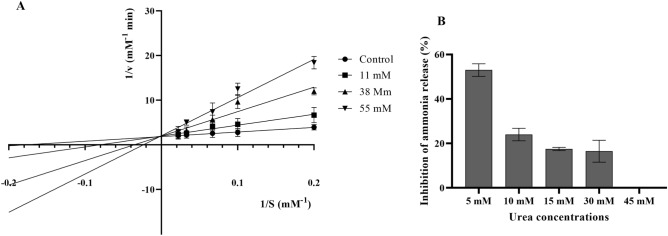
The Lineweaver–Burk plot showing the competitive inhibition of urease-catalysed hydrolysis of urea by different concentrations of lactic acid (**A**). Influence of increased concentrations of urea on urease activity in the presence of 38 mM lactic acid; data are presented as inhibition percentage of ammonia release (**B**); zero value means no inhibition of urease activity. The Michaelis–Menten plot of the predicted reaction rate of urea hydrolysis by urease as a function of a substrate concentration is shown in Fig. S1 (Supplementary Material).

The IC_50_ value of lactic acid was 38 mM ± 0.45 mM. Therefore, we used this concentration as well as IC_25_ (11 mM) and IC_75_ (55 mM) in the urease inhibition assay. Lactic acid inhibited urease activity in a dose-dependent manner. The Lineweaver–Burk plot showed that lactic acid interacted with a catalytic site of the enzyme, directly competing with the substrate (Fig. 6A). The type of inhibition was indicated by changes in enzyme kinetic parameters, of which the Michaelis–Menten constant (K_m_) increased with the increase of the inhibitor concentration, while the maximum reaction (V_max_) rate remained essentially unchanged (Fig. 6A). Specifically, K_m_ without lactic acid was 5.06 ± 1.2 mM and V_max_ 0.45 ± 0.06 mM/min, K_m_ 6.6 ± 1.18 mM and V_max_ 0.45 ± 0.07 mM/min for lactic acid 11 mM, K_m_ 8.9 ± 1.3 mM, V_max_ 0.44 ± 0.07 mM/min for lactic acid 38 mM and K_m_ 10.88 ± 0.8 mM, V_max_ 0.45 ± 0.06 mM/min for lactic acid 55 mM. The K_m_ value increase indicated decreased affinity of urea to the active site of urease in the presence of lactic acid. However, as shown in Fig. 6B, increased concentrations of the substrate (urea) neutralized the effect of the inhibitor (lactic acid), which clearly indicates the mechanism of competitive inhibition.

### Docking study

**Figure 7:**
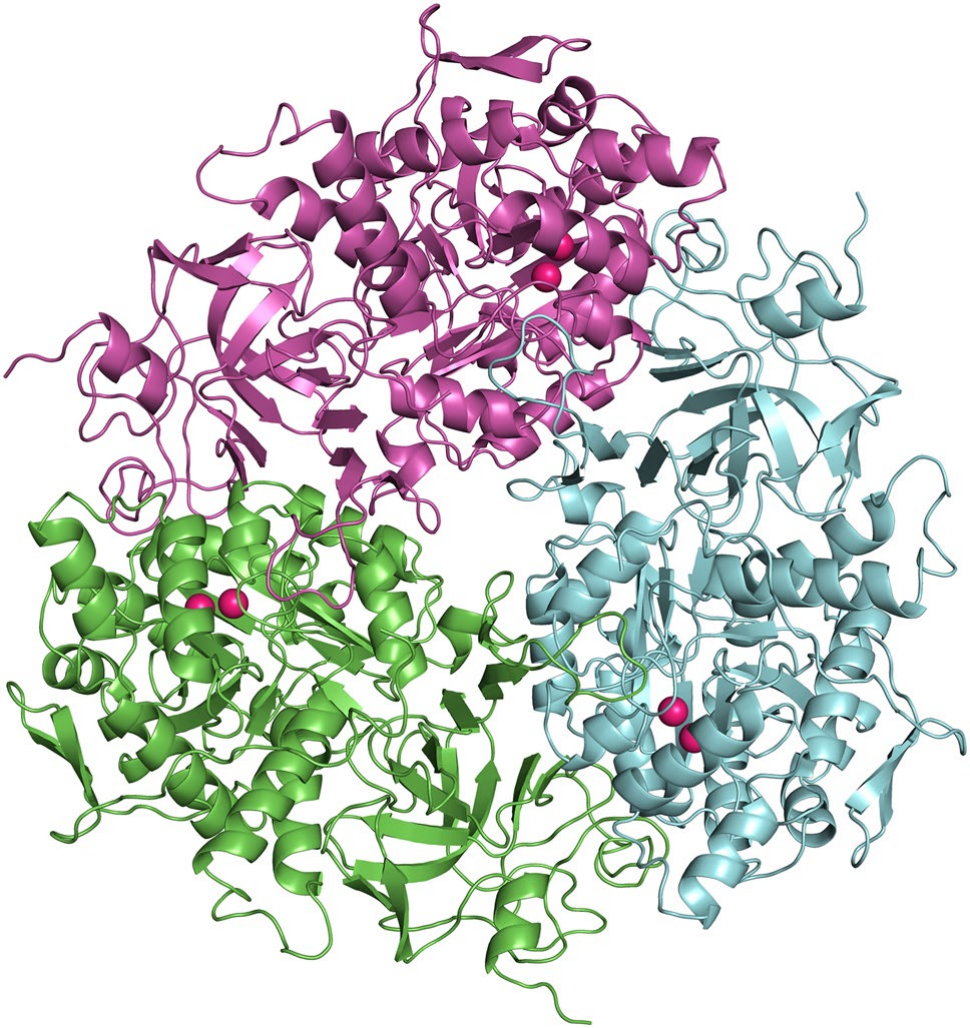
The three-subunit structure of *P. mirabilis* urease (homo-trimer) built using homology modeling methods; nickel ions in an active site of subunits are marked in red; three subunits are in different colors.

The structure of *P. mirabilis* urease was generated by the homology modeling method using the crystallographic structure of the Jack bean urease as a template (PDB 4gy7), and lactic acid (LA) was docked as a ligand, as described in the Materials and Methods. The model structure of the three subunits of *P. mirabilis* urease as a homo-trimer, binding six nickel ions in an active center is presented in Fig. 7. The two Ni ions were complexed by the linear and triagonal bonds of His residues (His246 and His272, and His134, His136) and Asp360 in the active center, which are stabilized by hydrophobic interactions and hydrogen bonds of N6-carboxylated Lys (KCX) (Table S1 and Fig. S2 Supplementary Material).

**Figure 8:**
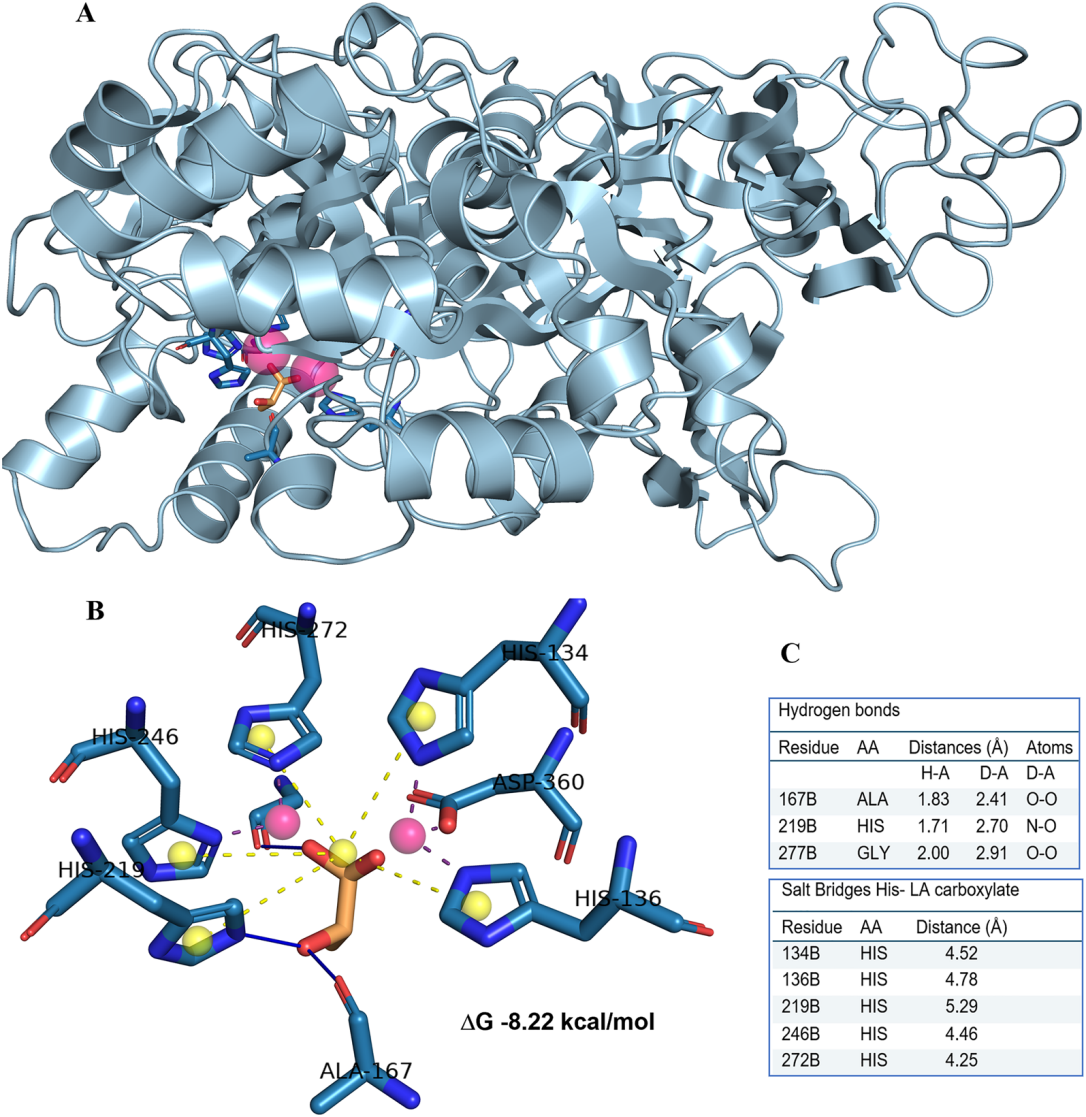
(**A**) Lactic acid (LA, in orange) docked to the catalytic center of subunit alpha of *P. mirabilis* urease located in the vicinity of two nickel ions (pink spheres). (**B**) The best docking pose of LA (orange) interacting with amino acids (blue tubes) in the active center of *P. mirabilis* urease, binding energy ΔG −8.22 kcal/mol (estimated *Ki *0.94 μM); hydrogen bonds between LA and His219, Ala167 and Gly277 are visualized as solid lines; long-range interactions, salt bridges, between lactic acid carboxyl and imidazole groups of five histidine residues are marked as dashed yellow lines; yellow spheres are charge centers in imidazole rings and LA carboxyl; complexation of Ni ions (pink balls) are marked as dark dotted lines; hydrogen atoms are omitted for clarity. Structures were analyzed and visualized in the PLIP 2.2.0 and PyMOL 2.3.4 software. (**C**) A table with listed amino acids interacting with LA through hydrogen bond interaction and salt bridges; calculations in PLIP 2.2.0 software.

Flexible docking showed the best docking position of LA in the catalytic center of *P. mirabilis* ureases in close proximity to two Ni ions and in the vicinity of the histidine residues involved in forming of salt bridges between the LA carboxylate and imidazole ring of His residues (Fig. 8A–C), or hydrogen bonds, similar to the Jack bean urease (Table S2). Two of the three residues forming hydrogen bonds with LA also participated in binding of urea (His219 and Gly277, Table S2 and Table S3). His136 involved in hydrogen bond with urea (Table S3), formed a salt bridge with LA (Table S3 and Fig. 8B, C). The estimated free energy of binding (ΔG, kcal/mol) was taken as a docking score (Autodock4.2.6). Comparison of docking of lactic acid (−8.22 kcal/mol) *versus* urea (−5.40 kcal/mol) revealed a remarkably better interaction of LA with an active center of *P. mirabilis* urease, as in the case of the Jack bean urease.

## Discussion

Urinary tracts of healthy women and men are inhabited by a diverse microbiota where the dominant species are microorganisms belonging to the genus *Lactobacillus*. They play a significant role in maintaining the homeostasis of this environment and the disturbance of which could affect the development of many uropathogens^15,39^. Many recent studies focus on the antibacterial and antibiofilm properties of natural microbiota of the urinary tract against the most common UTI pathogens such as: *Escherichia coli,* which cause approximately 80% of UTIs^40,41^, *Proteus mirabilis*^14,42^ or *Klebsiella pneumoniae*^43,44^. Additionally, a lot of scientific works concentrate on the practical use of selected *Lactobacillus* strains to UTIs treatment. Clinical testing such as that performed by Stapleton et al. showed that a probiotic containing *Lactobacillus* strains reduces the rate of UTI recurrence even by half ^45^. While most previous studies focused on *Lactobacillus* isolated from food, genital tracts or gut and their influence on UTI and comorbidities^45–47^, our findings concerned the strains isolated from human urine and their impact on the development of urinary stones caused by bacteria. We designed our work in such a manner as to imitate the environment of the human urinary tract in order to understand how these strains influence each other, which makes this study unique. Infectious urolithiasis is one of the most common urological diseases, with an increasing morbidity rate and ineffective therapy. Due to the difficulties in the treatment and the lack of effective preventive measures for this disease, researchers have recently focused on finding alternative treatment opportunities. Research conducted by Smanthong et al.^48^, about the *Sida acuta* Burm. F. ethanolic leaf extract, indicated that this extract has anti-struvite crystals properties, which makes it a potential new treatment agent. Other scientific works focused on the possibility of using compounds such as trisodium citrate^49^ or herbal extracts^50^.

These facts encouraged us to investigate the impact of natural microbiota of the urinary tract on the development of urinary stones caused by *P. mirabilis*. On the basis of the results of the present study, it can be concluded that some of the tested *Lactobacillus* strains are able to inhibit the crystallization of urine components. In the course of the experiments, the parameters that testify the intensity of crystallization, such as pH, ammonia release, concentration of Mg^2+^ and Ca^2+^ ions, *P. mirabilis* viability were assessed. The crystals were observed using phase-contrast microscopy. Among all the tested strains, *L. gasseri* stood out the most, showing the greatest inhibitory properties in opposition to strain *L. jensenii*. We observed that pH, release of ammonia, concentration of Ca^2+^ and Mg^2+^ ions were significantly lower, up to 8 h of incubation in mixed samples with *L. gasseri* compared to the control samples. In our previous study we found that *Lactobacillus* strains had antibacterial properties and abilities to inhibit the growth of *Proteus* strains at the level of up to 100%, mainly through secreted organic acids. *L. gasseri* showed the greatest antibacterial activity (72–97%) against many *P. mirabilis* strains (including those tested in this paper), while *L. jensenii* 22.2 exhibited the weakest properties^14^. Therefore, in the current study, the effect of these strains on the viability of *Proteus* bacteria in the environment of synthetic urine was also tested. It was assumed that this may be one of the mechanisms used by *Lactobacillus* strains to inhibit the crystallization process. Indeed, inhibition of *P. mirabilis* viability was observed in some tested samples. The strongest properties were noted in the samples with *L. gasseri* and *P. mirabilis* 5628 and 608/221 strains. In contrast to these results, the studies conducted by Torzewska et al.^47^ on the effect of *Lactobacillus* strains isolated from food on the crystallization caused by *P. mirabilis* demonstrated that tested *P. mirabilis* strains showed better viability in co-culture with *Lactobacillus*, which intensified the process of crystallization. This suggests that *Lactobacillus* strains isolated from the urinary tract might have some specific properties which they developed in the particular environment. The above results, indicating that some of the tested *Lactobacillus* strains are able to inhibit the crystallization process, were confirmed using a phase-contrast microscope. After 6 h of incubation, a lower amount or lack of struvite and apatite crystals were observed in the samples with *L. gasseri* and *L. crispatus* strains. Struvite crystals are formed at pH above 7.5 and can take various morphology depending on many factors, including pH. There are coffin-shaped crystals (like those observed in our research) and X-shaped dendrite crystals, formed due to a rapid increase in the pH value^51^.

Our previous work^14^ concerned organic acids secreted by lactic acid bacteria and their antibacterial properties. We found that all of the tested *Lactobacillus* strains used in that study produced lactic and succinic acid, however *L. jensenii* was distinguished by producing the highest concentration of succinic acid and *L. crispatus* 1.2 and *L.crispatus* 4 of lactic acid. Due to the fact that for some *P. mirabilis* strains the inhibition of their viability was not observed (although the suppression of crystallization occurred), the influence of lactic acid on urease activity was investigated.

*Lactobacillus* strains inhibited the crystallization process even in the presence of urease without *P. mirabilis* (Fig. 5B) and this phenomenon was also observed with different concentrations of lactic acid alone (Fig. 5C). In order to confirm the hypothesis that lactic acid inhibits the crystallization process through a direct interaction with urease, we performed the enzyme activity inhibition test. So far, there are no literature data describing the mechanism of inhibiting the urease activity by lactic acid. However, it has been shown that organic acids can bind many trace metals, including nickel which is a part of the active site of urease^52^. *P. mirabilis* urease is metalloprotease, built of three subunits with a total mass of 200–700 kDa, containing a lot of cysteine and histidine residues and, as already mentioned, nickel atoms in the catalytic center. Many other microorganisms produce this enzyme and it is an important factor in the pathogenicity of these bacteria^53^. In the course of our experiments, to determine the lactic acid inhibitory activity, urease from Jack bean (*Canavalia ensiformis*) was used as a reference enzyme. Kinetic parameters of urease activity K_m_, and V_max_, revealed that lactic acid acts as a competitive inhibitor which binds to the active site of the enzyme. The docking experiments confirmed that lactic acid binds to the catalytic center of enzyme like competitive inhibitors, with remarkably higher affinity than urea did (scores −8 kcal/mol vs. −5 kcal/mol), which confirmed its inhibitory potency as a good ligand. In this context, lactic acid can be considered as a modulator of urease activity with pharmacological potential. Interestingly, the interaction between lactic acid and urea may involve the same histidine residues that bind urea, but in addition to hydrogen bonds (as in the case of urea), salt bridges may also be formed, stabilizing the position of lactic acid in the catalytic center of the enzyme in the ticks of histidine residues. In turn, as demonstrated by our in vitro study, the competing effect of high urea concentrations on the lactic acid residues in the enzymatic center may lead to displacing the lactic acid molecule from the site of its binding in urease. Based on in silico studies, we suggest that long-range salt bridges may be disrupted by high concentrations of urea.

To date, various types of urease inhibitors have been identified, e.g. hydroxamic acids, phosphoramidates, quinones, polyphenols or heterocyclic compounds^54^. Acetohydroxamic acid (AHA) is recommended in supporting the treatment of urolithiasis. However, therapeutic application of AHA has limitations due to many side effects like risk of hemolytic anemia or leukopenia^55^. Effective and safe urease inhibitors would be a breakthrough in the treatment of urolithiasis caused by urease-producing bacteria. Therefore, many researchers struggle with this issue, such as Milo S. et al.^56^, who pointed out the effectiveness of another low molecular weight organic acid like 2-MA as a safer alternative to the currently used AHA acid.

## Conclusion

The results of our study indicate that *Lactobacillus* strains that inhabit the urinary tract are able to suppress the crystallization process, i.e. one of the initial stages of the urinary stones formation. In the course of the presented studies, we showed that their antagonistic effect is multidirectional, and in addition to the antibacterial properties against *P. mirabilis*, lactic acid produced by them affects the activity of urease through interaction with the catalytic domain of the enzyme. These studies are preliminary and their continuation on an in vivo model will confirm the usefulness of this lactic acid as a factor supporting the treatment of urolithiasis.

### Supplementary Information

Supplementary Information.

## Data Availability

All data generated or analyzed during this study are included in this published article and its supplementary information files.
