# Supplementary material for: Antibacterial properties and urease suppression ability of Lactobacillus inhibit the development of infectious urinary stones caused by Proteus mirabilis
Source: Sci Rep. 2024 Jan 10;14:943. doi: 10.1038/s41598-024-51323-0 (PMC10781950; doi:10.1038/s41598-024-51323-0)
Supplement: Supplementary file 1 — Supplementary Information. [file 41598_2024_51323_MOESM1_ESM.docx]

**Supplementary Information**

Title: Antibacterial properties and urease suppression ability of *Lactobacillus i*nhibit the development of infectious urinary stones caused by *Proteus mirabilis*

Authors: Dominika Szczerbiec, Katarzyna Bednarska-Szczepaniak, Agnieszka Torzewska


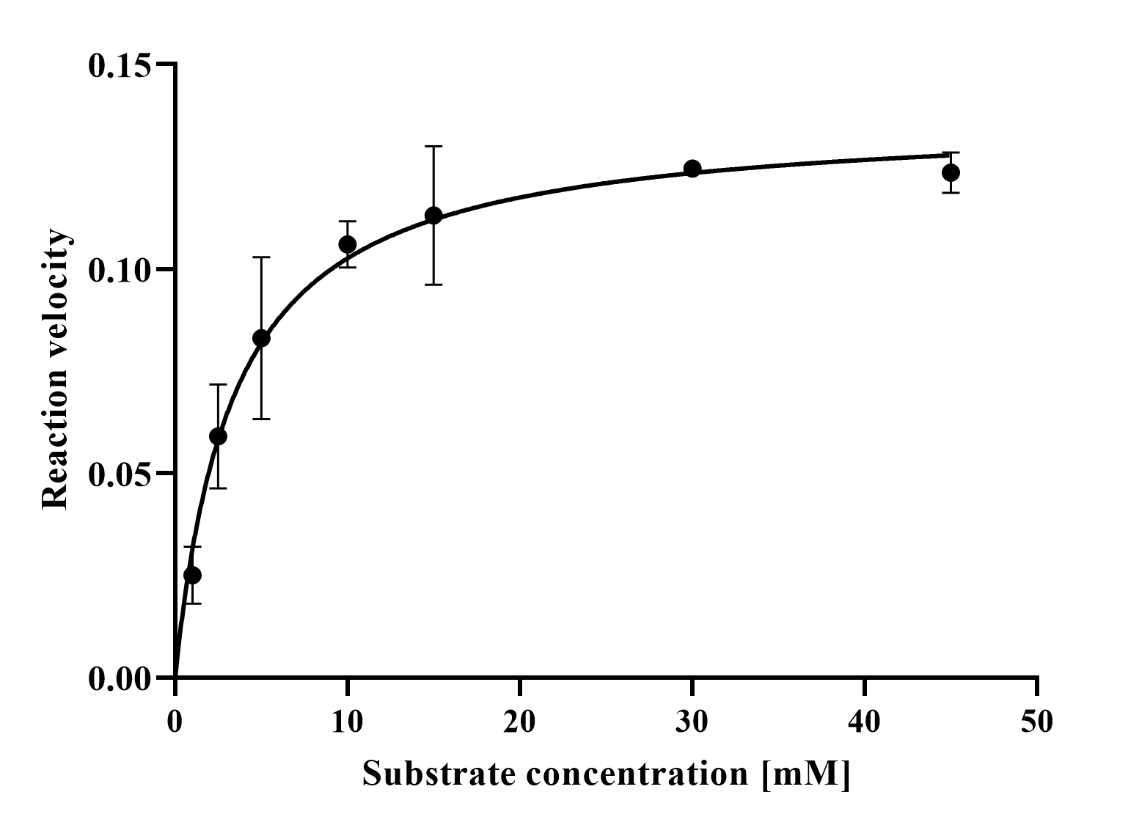


Fig. S1. Michaelis-Menten kinetics of Jack bean urease, with the substrate urea. The results are presented as mean ± standard deviation (SD) of three experiments.

Tab. S1. Metal complexes formed by two Ni ions and amino acids in catalytic centers of *P. mirabilis* urease (model) and Jack bean (*Canavalia ensiformis*) urease (PDB 4gy7), predicted by PLIP.

|  | *Proteus mirabilis* | | | *Canavalia ensiformis* | | |
| --- | --- | --- | --- | --- | --- | --- |
| Metal Complexes | Residue | AA | Distance (Å) | Residue | AA | Distance (Å) |
| Ni, linear |  |  |  |  |  |  |
| 1 | 246B | HIS | 1.95 | 519B | HIS | 2.08 |
| 2 | 272B | HIS | 1.99 | 545B | HIS | 2.08 |
| Ni, trigonal.pyramidal | |  |  |  |  |  |
| 1 | 134B | HIS | 2.17 | 407B | HIS | 2.11 |
| 2 | 136B | HIS | 2.00 | 409B | HIS | 2.06 |
| 3 | 360B | ASP | 1.89 | 633B | ASP | 2.05 |


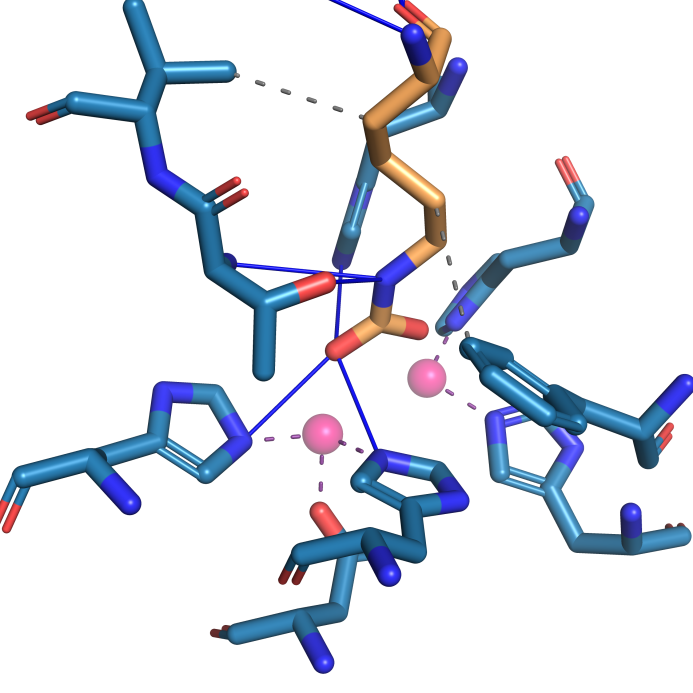


Fig. S2. Graphical representation of a catalytic center of *P. mirabilis* urease, complexes of Ni ions with amino acids are shown. The homology model of *P. mirabilis* urease was built on Jack bean urease crystallographic structure (PDB 4gy7, 1.49 Å resolution). His residues and Asp are marked in blue, orange – N6-carboxylated Lys, pink – Ni ions; dotted lines –hydrophobic interactions; dashed lines at Ni atoms – metal complexation; solid lines – hydrogen bonds; hydrogen atoms are omitted for clarity.

Tab. S2. Lactic acid (LA) interacting with amino acids of *P. mirabilis* and *Canavalia ensiformis* urease catalytic centers. Five His residues (*P.*m His134, His136, His219, His246, His272) form salt bridges with LA carboxylate.

| *Proteus mirabilis*  Hydrogen bonds | | | | | *Canavalia ensiformis*  Hydrogen bonds | | | | |
| --- | --- | --- | --- | --- | --- | --- | --- | --- | --- |
| Residue | AA | Distances (Å) | | Atoms | Residue | AA | Distances (Å) | | Atoms |
|  |  | H-A | D-A | D-A |  |  | H-A | D-A | D-A |
| 167B | ALA | 1.83 | 2.41 | O-O | 440B | ALA | 1.93 | 2.51 | O-O |
| 219B | HIS | 1.71 | 2.70 | N-O | 492B | HIS | 1.88 | 2.82 | N-O |
| 277B | GLY | 2.00 | 2.91 | O-O | 519B | HIS | 3.55 | 3.93 | N-O |
|  |  |  |  |  | 550B | GLY | 1.83 | 2.69 | O-O |
| Salt Bridges His- LA carboxylate | | | | | Salt Bridges His- LA carboxylate | | | | |
| Residue | AA | Distance (Å) | |  | Residue | AA | Distance (Å) | |  |
| 134B | HIS | 4.52 | |  | 407B | HIS | 4.43 | |  |
| 136B | HIS | 4.78 | |  | 409B | HIS | 4.93 | |  |
| 219B | HIS | 5.29 | |  | 492B | HIS | 5.35 | |  |
| 246B | HIS | 4.46 | |  | 519B | HIS | 4.60 | |  |
| 272B | HIS | 4.25 | |  | 545B | HIS | 4.28 | |  |

H – hydrogen atoms; A – acceptor, D – donor.

Tab. S3. Urea forming hydrogen bonds with amino acids of *P. mirabilis* and *Canavalia ensiformis* urease catalytic centers.

| *Proteus mirabilis*  Hydrogen bonds | | | | | *Canavalia ensiformis*  Hydrogen bonds | | | | |
| --- | --- | --- | --- | --- | --- | --- | --- | --- | --- |
| Residue | AA | Distances (Å) | | Atoms | Residue | AA | Distances (Å) | | Atoms |
| 136B | HIS | 2.48 | 2.98 | N-N | 407B | HIS | 2.7 | 3.6 | N-N |
| 219B | HIS | 3 | 3.74 | N-O | 409B | HIS | 2.17 | 2.8 | N-N |
| 277B | GLY | 2.18 | 3.16 | N-O | 492B | HIS | 2.35 | 3.23 | N-O |
| 363B | ALA | 2.17 | 2.97 | N-O | 519B | HIS | 1.87 | 2.65 | N-O |
|  |  |  |  |  | 545B | HIS | 2.36 | 3.23 | N-O |
|  |  |  |  |  | 636B | ALA | 2.16 | 3.1 | N-O |
|  |  |  |  |  | 636B | ALA | 2.74 | 3.57 | N-O |
|  |  |  |  |  |  |  |  |  |  |
